# The Sick Room

**Published:** 1883-12

**Authors:** 


### THE SICK ROOM.

Ah, there is a bird's shadow flitting across the pane. The tree-top sways and trembles with soft rustlings, a white cloud floats dreamily over the blue, and now—O delight and wonder!—the bird himself comes in sight and perches visibly on the bough, dressing his feathers and quivering forth a few notes of song. All the world, then, is not lying in bed because we are, is not tired of its surrounding, has not the back-ache. What a refreshing thought! And though this glimpse of another life, the fresh natural life from which we are shut out—that life which has nothing to do with pills and potions, tiptoe movements, whispers, and doctors' boots creaking in the entry—may cause the hot tears to rush suddenly into our eyes, it does us good, and we begin to say with a certain tremulous thrill of hope: "When I go out again I shall do so and so."

Ah, if nurses, if friends knew how irksome, how positively harmful is the sameness of a sick-room, surely love and skill would devise remedies. If it were only bringing in a blue flower to-day and a pink one to-morrow, hanging a fresh picture to vary the monotony of the wall, or even an old one in a new place—something, anything—it is such an infinite relief. Small things and single things suffice. To see many of his surroundings changed at once confuses an invalid; to have one little novelty at a time to vary the point of observation stimulates and cheers. Give him that, and you do more and better than if you filled the apartment with fresh objects.

It is supposed by many that flowers should be carefully kept away from sick people; that they exhaust the air or communicate to it some harmful quality. This may, in a degree, be true of such strong, fragrant blossoms as lilacs or garden lilies, but of the more delicately-scented ones no such effect need be apprehended. A well-aired room will never be made close or unwholesome by a nosegay of roses, mignonette or violets, and the subtle cheer which they bring with them is infinitely reviving to weary eyes and depressed spirits.—*Home and Society.*

